# Infectious Bronchitis Virus Variants: Molecular Analysis and Pathogenicity Investigation

**DOI:** 10.3390/ijms18102030

**Published:** 2017-09-22

**Authors:** Shu-Yi Lin, Hui-Wen Chen

**Affiliations:** 1 Department of Veterinary Medicine, National Taiwan University, Taipei 10617, Taiwan; susielin531@gmail.com; 2 Research Center for Nanotechnology and Infectious Diseases, Taipei 11529, Taiwan

**Keywords:** infectious bronchitis virus, variants, genotypes, antigenicity, pathogenicity

## Abstract

Infectious bronchitis virus (IBV) variants constantly emerge and pose economic threats to poultry farms worldwide. Numerous studies on the molecular and pathogenic characterization of IBV variants have been performed between 2007 and 2017, which we have reviewed herein. We noted that viral genetic mutations and recombination events commonly gave rise to distinct IBV genotypes, serotypes and pathotypes. In addition to characterizing the *S1* genes, full viral genomic sequencing, comprehensive antigenicity, and pathogenicity studies on emerging variants have advanced our understanding of IBV infections, which is valuable for developing countermeasures against IBV field outbreaks. This review of IBV variants provides practical value for understanding their phylogenetic relationships and epidemiology from both regional and worldwide viewpoints.

## 1. Introduction

Infectious bronchitis virus (IBV) is an economically important pathogen in the poultry industry. Infectious bronchitis, resulting from IBV, is an acute, highly contagious infection accompanied by upper respiratory symptoms and urinary and reproductive system failures, resulting in low production rates, egg drop, and death in chickens. Since its first documentation in 1931 in the United States [1], IBV has spread to all continents that host chicken farming. Vaccination is the most common method to prevent IBV infections in chickens, and commercial vaccines include live attenuated vaccines and inactivated vaccines. However, the cross protections of the vaccines are limited, as novel serotypes are continuously emerging, and immune failures are reported frequently. The Massachusetts (Mass) and 4/91 (also known as 793B) types are widely used around the world, while local strains have been selected for vaccine development in individual regions.

IBV is a *gammacoronavirus* belonging to the *Coronaviridae* family, and its virion contains a single copy of a positive-sense, single-stranded 27.6-kb RNA. The IBV genome encodes four structural proteins, polyproteins 1a and 1ab, and several accessory proteins. The coding regions of the polyproteins cover two-thirds of the viral genome and are subjected to further processing into 15 non-structural proteins (nsp2–16). Accessory proteins include 3a, 3b, 5a, and 5b, but these may be missing in some IBV strains. The structural proteins are spike (S), envelope (E), membrane (M) and nucleocapsid (N). The S protein, especially the S1 subunit that is formed by post-translational cleavage [2,3], has proven to be critical for antigenic neutralization, hemagglutination and cell tropism determination [4,5,6]. Most previously published IBV molecular characterizations have focused on the analysis of the *S* gene, which may not be sufficient to explain the observed changes in the serotypes and pathotypes of IBV variants. Several studies have noted that viral replication and immune escape may be modulated by non-structural and accessory viral proteins in IBV and other coronaviruses, such as mouse hepatitis virus and severe acute respiratory syndrome coronavirus (SARS-CoV) [7,8]. Additionally, cytotoxic T lymphocyte epitopes in the N protein can protect chickens from IBV infection [9,10]. These observations emphasize the importance of studying the full-length IBV genome and its associations with serological and pathogenic characteristics. To provide a comprehensive outlook on circulating IBV variants, we have reviewed numerous studies reporting IBV variants between 2007 and 2017 from different continents, and summarized the findings from molecular analyses, epidemiological data, and antigenicity/pathogenicity investigations (Table 1).

## 2. IBV Classification

Typically, IBV can be classified by nucleic acid-based or antibody-based methods, which provides genotyping and serotyping results, respectively. The cross neutralization test, a conventional method for distinguishing viral serotypes, analyzes the neutralizing activity of antisera prepared in specific-pathogen-free (SPF) chickens. Archetti and Horsfall Jr [11] demonstrated the calculation of the relatedness value (*R*-value) obtained from neutralization tests. The hemagglutination inhibition test is also used in serotyping IBV, but disadvantageously, the virus must be treated with neuraminidase to yield hemagglutination activity [12]. The enzyme-linked immunosorbent assay (ELISA), in contrast, cannot discriminate between different serotypes, as the cross-reactive antibody interferes with the detecting signal, particularly when the coating antigen is derived from whole virions. To this end, several serotyping ELISAs incorporating the specificity of monoclonal antibodies have been reported [13,14]. Collectively, performing serotyping procedures with embryonic eggs or chickens is not cost-effective; therefore, genotyping, which is relatively simple to perform, has become increasingly popular with the development of reverse transcriptase polymerase chain reaction (RT-PCR) techniques, gene sequencing technology, and bioinformatics. Most laboratories use these tools in a combined method to amplify the *S1* gene of IBV variants, and then analyze the sequences using Basic Local Alignment Search Tool (BLAST) or phylogenetic analyses. In addition, using genomic sequence comparison, recombination events can be detected among two or more nucleotide sequences, and the breakpoints can be identified by the Recombination Analysis Tool (RAT) or SimPlot [15]. While the S1 sequence is reportedly strongly correlated with the protective R value [16], changes in serotype can result from only a few changes in the amino acids of the S1 protein [17]. However, there was no unifying nomenclature on IBV genotypes, leading to distinctive and confusing names based primarily on the viruses’ geographic origin. To provide a more unified IBV genetic classification, Valastro et al. [18] put forward a method that defines IBV strains into six genotypes comprising 32 distinct viral lineages based on the complete *S1* gene. In the Table 1, the genotype and lineage of each strain according to this classification method were indicated together with the traditional sorting name. IBV pathotypes can vary greatly among strains, and clinical symptoms caused by infection can be diverse due to various chicken breeds, environmental circumstances and immunity levels. Although IBV demonstrates clear tropism by producing lesions in the respiratory system, kidney, reproductive system, and alimentary tract, the viral pathotypes observed in field outbreaks are usually more complicated due to co-infections with other pathogens [19]. Thus, pathogenicity results generated from experimental infection using SPF chickens with good laboratory practice are more reliable. The protectotype, a direct indicator of the protective efficacy against a virus, is a practical typing method for vaccine evaluation. However, the large number of experimental animals and labor required to determine the pathotypes or protectotypes of IBV are costly.

## 3. Evolution Mechanism of IBV

According to modern evolutionary hypotheses [47], genetic diversity and selection processes are the two basic steps of evolution. Spontaneous mistakes (nucleotide substitutions, deletions or insertions, and recombination) made during viral replication provide the materials for selection, eventually resulting in virus evolution. The mutation rate of RNA virus is 10^−4^ to 10^−5^ substitutions per nucleotide (nt) per round of replication. Even though a 3′ to 5′ exoribonuclease (ExoN) in coronavirus nsp14 was proven to have RNA proofreading function, the mutation rate remains significantly higher than the likes of DNA genome organisms, such as *E. coli*, which has a mutation rate of only 10^−9^ to 10^−11^ substitutions per nt per round of replication [48,49,50]. However, the proofreading function of nsp14 may also play an important role in coronavirus replication, to reach a balance between fidelity and diversity, facilitating adaptation to environmental selection pressure. Details regarding IBV selection regulation were fully elucidated by Toro et al. [51], and selection forces may be immune responses induced by several types of vaccines, the microenvironment of infected hosts, or physical and biosafety conditions. In chickens, immune responses can be ceased by infection with an immunosuppressive virus, such as chicken anemia virus, infectious bursal disease virus, and Marek’s disease virus. Additionally, viral immunity levels and specific antibodies may differ across farms or countries based on their imposed immunization schedules. Host microenvironments can have slight differences, even though in individual hosts, distinct tissues have distinct cell receptors, temperatures, pH values, enzymes, and concentrations that can alter the predominant virus genotype by selective pressure [52,53]. Physical conditions aiding virus spread include environmental temperature, humidity, and wind. The distance of a chicken farm from an infectious source and the all-in-all-out system play important roles in biosafety conditions. While numerous IBV variants have emerged because of errors during genomic replication, only a few variants became endemic in specific regions under these described selection factors, while the others inflicted no long-term effects. This theory can also explain why some strains are first observed in one county, disappear, and then reappear as an endemic in other countries several years later. Zhao et al. [54], confirmed that every IBV-encoding gene undergoes positive selection, and analyzed the evolution rate of IBV in China, finding that the *E* gene evolves at the fastest rate among all the structural protein coding genes, with a substitution rate of 10^−6^ nt substitutions per site per year. The *N* gene evolves the slowest, with 10^−5^ nt substitutions per site per year. These selective pressures have forced IBV to rapidly evolve in the past several decades, and variants having distinct genotypes, serotypes, and pathogenic types have been continually reported.

## 4. Asia

IBV in Taiwan was first documented in 1958, and it can be divided into two groups, Taiwan group I (TW-I) and Taiwan group II (TW-II). Both groups are distinct from other strains around the world in terms of their genotypes and serotypes [55,56]. Except for the *nucleocapsid* gene, the structural and non-structural genes of Taiwan isolates are more closely related to China strains than US strains [57]. While most Chinese neighbors have been affected by Chinese QX-like IBV strains, this has not occurred in Taiwan. However, the Chinese CK/CH/LDL/97I (Q1-like) recombinant IBV strain was reported in Taiwan by Chen et al. [58]. The surveillance program conducted in poultry slaughterhouses showed IBV prevalence rates at 17% during 2005–2006 and 39% in 2013, respectively, from which several variants derived from recombination among Taiwan, Chinese Q1, Mass and Japan types were detected [59,60]. The Q1-like and Japan-like IBV strains revealed high lethality and possessed multi-organ tropism in experimentally infected chickens (unpublished data). In addition to recombination events, point mutations in a TW-I strain were found in the IBV 3575/08, resulting in increased pathogenicity and serotype and immunotype changes, suggesting that IBV in Taiwan is continuously evolving [34].

Because China is a large country and large populations of chickens are maintained at high densities in large-scale factories or backyards, IBV strains have numerous opportunities to spread and recombine with each other. In China, IBV was first observed in the early 1980s, and IBV outbreaks have since been frequently reported. According to the phylogenetic analysis of new isolates based on the *S1* gene, the predominant strains in China are the QX-like strains [61]. The IBV QX strain was first isolated in China in 1996 and was characterized by proventriculus swelling in affected chickens [62]. Until 2009, the QX-like strain was found to be the second most prevalent strain in China [63]. In fact, the proportion of QX-like isolates has increased from 20% in the 2000s to over 60% since 2007 [54]. Han et al. [64] reported that 54.1% (119/220) of IBV isolates in China were of the LX4 type, which is a well-known QX-like type IBV. The LDT3 and 4/91 (also known as 793B) types have also frequently been isolated in China recently [65,66,67]. The N-terminal sequence of the S1 protein of the 4/91 type is reportedly more variable than those of other types, such as Mass and QX [68].

Commercial vaccines used against IBV in China include the attenuated live strains H120, LDT3, 4/91 and some other inactivated vaccines, such as M41. Mass type is the most widely inoculated vaccine and also circulates in China, as its isolation rates range from 6.94% to 8.64%, according to previous studies [23,64]. Because of recombination with other strains, some Mass type isolates can be cross-neutralized by H120 antiserum and are completely protected against by the H120 vaccination. However, two isolated Mass type recombinant strains, CK/CH/LDL/110931 and CK/CH/LHB/130573, are serologically different from the H120 vaccine and thus cannot be protected against [23]. Several amino acid point mutations in the S1 subunit may be responsible for this immune escape [64].

With plentiful local and vaccine strains simultaneously circulating in China, recombination has become a common phenomenon, and recombination events within vaccine strains [23,69,70] and other local strains [70,71] are widely found. For example, the CK/CH/LGX/130530 strain has a breakpoint in nsp14, and its 5′-terminal portion is from H120, while its 3′-terminal portion was donated by the tl/CH/LDT3/03-like virus. The lower virulence of CK/CH/LGX/130530 also provided evidence that non-structural proteins play an important role in virulence determination [29]. The Sczy3 strain is a recombinant strain that was derived from the major parental LX4 strain and the minor parental H120 strain [32]. The nephropathogenic strain SAIBK, which also recombined from the major parent SC021202 and H120, was determined to be the 4/91 serotype [33]. The CK/CH/LSD/100408 strain has sequences from both LX4 and tl/CH/LDT3/03 type strains in different parts of its *S1* gene [61]. A newly emerged genotype was classified as lineage GI-28 since it was found to be genetically different from other IBV strains. The representative strains are CK/CH/LGX/111119 and CK/CH/2010/JT-1, which are recombinant strains originated from LX4 or partridge/GD/S14/2003 [20,21].

Taiwan groups (TW-I and TW-II) of IBV strains have been isolated in China and the emergence of TW types strains has increased since 2009 [26,65,72,73]. Xu et al. [26] reported a highly virulent recombinant created from *S1* genes from the QX and TW-I strains, named the GD strain, that can induce severe respiratory symptoms, renal lesions, and mortality in approximately 40% of cases. The GD strain was classified as the TW genotype, but it can be completely protected by the QX-like IBV strain JS [27]. Another TW-I and LX4 strain recombinant (CK/CH/LDL/140520) that causes cystic oviducts as well as nephritis and respiratory distress in one-day-old chickens was identified by Gao et al. [28]. Moreover, the TW-II-like strain CK/CH/LHB/100801 was found to have emerged in chicken flocks in China in 2011, but this strain exhibits mutations and deletions from the strain isolated in Taiwan [69].

In Japan, IBV can be divided into three genetic groups, JP-I, JP-II and JP-III. In addition to these groups, some 4/91 type strains were isolated and confirmed to be variants [74,75]. JP/Wakayama/2003 is a 4/91 type strain that can be cross-neutralized by serum from 4/91, JP-I (GN strain) and H120, with R-values higher than 79. Another 4/91 type strain, JP/Iwate/2005, is completely protected by the 4/91 vaccine and the JP-II strain (TM-86w), even though its R-value with TM-86w was low [35]. Notably, all these 4/91 type isolates are more virulent than the vaccine strain. A novel genotype strain, JP/Ibaraki/168-1/2009, was distinctly isolated with other Japanese strains but was similar to the TC07-2 strain that was isolated in south China based on the hypervariable S1 region, and serology tests also confirmed this strain as a novel serotype. This novel strain cannot be neutralized by Japan type strains or the H120, Gray and 4/91 strains [76].

Two groups of IBVs exist in Korea, named Korean group I and Korean group II. Korean group I is more closely related to the Mass type, while Korean group II is a distinctive branch. Among Korean group II viruses, three subgroups were found, QX-like, KM91-like and New Cluster 1 (NC1) [77]. All of the new isolates that emerged from 2005 to 2010 were nephropathogenic and clustered into Korean group II. Among these, K716/05 was found to be a KM91 and QX recombinant strain in the *S1* gene [77]. A KM91-like backbone recombinant, SNU8067, was reported to inhibit hierarchal ovarian follicle formation and oviduct maturation. Recombination with the Mass type vaccine strain has also been documented [36], and the NC1 strain is continuously evolving and accumulating point mutations [78].

In Thailand, while the vaccine strains derived from M41, H120, Ma5, Connecticut (Conn), 4/91 and the local DLD strain were widely used, three distinct groups including the unique Thailand type QX-like, and Mass type are now in circulation [79]. Promkuntod et al. [80] reported that 62.5% (15/24) of Thailand isolates are QX-like variants, demonstrating that QX-like strains have been predominant since 2009.

In India, Patel et al. [81] isolated a strain similar, but genetically distinct, to the Mass type vaccine M41 strain. The first 4/91 type strain in India was isolated by Sumi et al. [82], which may be a new vaccine strain variant based on its genomic sequence.

Ganapathy et al. [83] demonstrated that six IBV genotypes were detected in the Middle East from 2009–2014: 4/91, IS/1494/06, Mass, IS/885/00, Q1 and D274. In total, 32.87% and 18.87% of the *S1* genes of the 4/91 and Mass type strains, respectively, were more than 99% homologous with vaccine strains, indicating the genetic mutation of vaccine strains. The authors of this study also noted that 4/91 field strain isolates identified after 2012 were differently clustered with former isolates. Other researchers have reported the appearance of some China-like strains in the Middle East, such as strains similar to CK/CH/Guangdong/Xindadi/0903 [84] and CK/CH/LDL/97I [85] based on their S1 sequences. Interestingly, the LDL/97I-like strains that were first isolated in the Middle East exhibited more extensive tissue tropism, as they were detectable in trachea, kidneys, ovarian tissue, and cecal tonsils, than the original LDL/97I strain that was limited to the respiratory system and kidneys.

## 5. Africa

In Egypt, IBV isolates are mainly divided into the Egy/Var I, Egy/Var II and Mass type groups [38]. The Egy/Var I and Egy/Var II variants, which recombined from the original Egyptian variant and the Israeli strain, are grouped with Middle Eastern IBV strains. In addition, these variants showed high virulence in one-day-old SPF chickens, with 50% mortality. Furthermore, another study on Egy/Var type isolates identified a deletion at position 63, a substitution at I69A/S, and an additional *N*-glycosylation site in the S1 protein [11]. The first QX-like strains were confirmed in Zimbabwe in 2011 [86], and Italy 02 type strains were first detected in Africa (Morocco) by Fellahi et al. [87] in 2014. At that time, the proportion of IBV Italy 02 type strains was 32%, and they quickly became the second most prevalent genotype in Morocco. However, Moroccan Italy 02 isolates were slightly different from European Italy 02 strains based on their *S1* genes, according to phylogenetic tree analysis.

## 6. Europe

QX strains were first detected in Europe in the Netherlands between 2003 and 2004, and their proportionality has since sharply increased. Over the past decade, QX-like strains, characterized as European QX because of some nucleotide substitutions, have been detected in the United Kingdom, Finland, Hungary, Russia, Slovenia, Spain, Sweden and Switzerland [44,88,89,90,91,92,93,94]. Pohjola et al. [90] found a unique 117LDKG120 sequence that appeared to be a recombination between a QX strain and an unknown IBV strain in Finland, a country in which IBV is not vaccinated against. Abro et al. [88] dissected a 2010 isolated Swedish QX strain, CK/SWE/0658946/10, and found several regions, especially amino acids 1650–2850, 3735–3940 and 4915–5285 located in the *1ab* gene, that underwent strong positive selective pressure and had numerous non-synonymous substitutions. In Russia, three QX-like IBVs were found that contained recombinant *S1* genes from the vaccine strains H120, 4/91, and D274 [92].

The Q1 strain was initially isolated in China by Yu et al. [95] between 1996 and 1998 in young layers exhibiting proventriculitis and respiratory symptoms, but no kidney lesions. This strain did not circulate outside Asia but was detected in 15-day-old broiler chickens in Italy in 2011 [96]. Furthermore, Q1-like isolates in Italy targeted the proventriculus and kidney [42]. Another study analyzed the full-length genome of the Q1-like strain γCoV/Ck/Italy/I2022/13, which was isolated in Italy in 2013, and noted multiple recombination events [43]. Evidence clearly shows the circulation of variant Q1 strains in Italy.

As a country that does not vaccinate against IBV, Finland has been exempt from IBV clinical cases since the 1970s. However, several vaccine-like pathogenic strains, including the D274 and 4/91 strains, were found in a 2011 outbreak, indicating the variation of vaccine strains [90]. Finland is now considering improving their vaccination process with live attenuated vaccines.

According to Krapez et al. [91], the predominant IBV strains in Slovenia from 1990 to 2005 were 624/I type strains (9/15, 60%), which can be distinctly clustered with strains of earlier isolation periods and geographical origin, based on the *S1* gene.

## 7. America

Many types of commercial vaccine strains have originated from the United States, such as the Mass, Conn, and Arkansas (Ark) types. A nephropathogenic IBV strain, DMV/1639/11, isolated from Delmarva in 2011 was similar to the strain responsible for the Pennsylvania IBV outbreak from 1997 to 2000. When the Mass vaccine type was combined with the Conn or Ark types in a laboratory setting via the eye-drop method, the virus shedding rate and renal lesions in broiler chickens was decreased after DMV/1639/11 challenge. However, in the commercial setting of live vaccine spraying, trachea and kidney protection was not observed [97]. While the Cal99 strain usually causes only respiratory symptoms, a Cal99 variant was found to be nephropathogenic and could spread to respiratory and gastrointestinal tracts, and the bursa [40]. A novel IBV genotype in Georgia was determined to have *S1* gene sequences from both the Australian-isolated N1-62 strain and the Ark DPI strain [98].

The IBV strains in Canada can be divided into four groups: Canadian variants, vaccine-like, US variant-like, non-Canadian, and non-US. While the current Mass and Conn type vaccines can provide satisfactory protection results to most of the groups, the emergence and circulation of the 4/91 type indicates the need for a 4/91 type vaccine [99].

Brazil has reported the most IBV isolates in North America. Brazilian IBV strains form a unique Brazilian cluster that includes three subclusters, Brazil 01, 02 and 03 [100], but their genetic variation was low for a long time [101]. The D207, Mass, Conn, and Ark serotypes have also been found in Brazil [102], and the 4/91 strain was first documented by Villarreal et al. [100]. Analysis of IBV-positive rates in different tissues showed the highest presence of IBV in the digestive system (43.5%), followed by the respiratory system (37.7%) [103]. The proventriculus type Q1-like strain was found in other South American countries, such as Argentina and Uruguay [104].

## 8. Oceania

IBV evolution is independent in Australia because of its geographical isolation. IBV strains in Australia can be classified into three groups based on the *S1* gene: Australian group 1, containing vaccine and vaccine-like strains, group 2, emerging from the 1980s to the 1990s, and group 3, containing newly isolated recombinant strains from groups 1 and 2. In 2016, however, two group 2 strains were isolated that had been undetectable for two decades [45,46]. Quinteros et al. [46] proposed the existence of an unknown IBV parental strain circulating in the field, which explains how the 1980s strains underwent recombination to fit new environments. Former studies have found that the open reading frames (ORFs) of Australian group 2 IBV strains were analogous to those of turkey coronaviruses in that they lack the *3a*, *3b*, *5a* and *5b* genes. However, while the recently isolated group 2 strain had the *3b* and *5b* genes, it contained genetic mutations that could influence transcription [105].

## 9. Conclusions

Among the studies reporting the IBV variants reviewed herein, mutations and recombination can take place in both the structural and the non-structural proteins, although the exact link of these variations to pathogenicity alternation is still unknown. Most of the mutations and recombination events have been detected in the *S* gene (54%, 15 out of 28 references reviewed) and then in the polyprotein 1a and 1ab (35.7%, 10 out of 28 references reviewed). In particular, the nsp 2–6 were more variable in these studies, consistent with previous observations [54].

**Figure 1:**
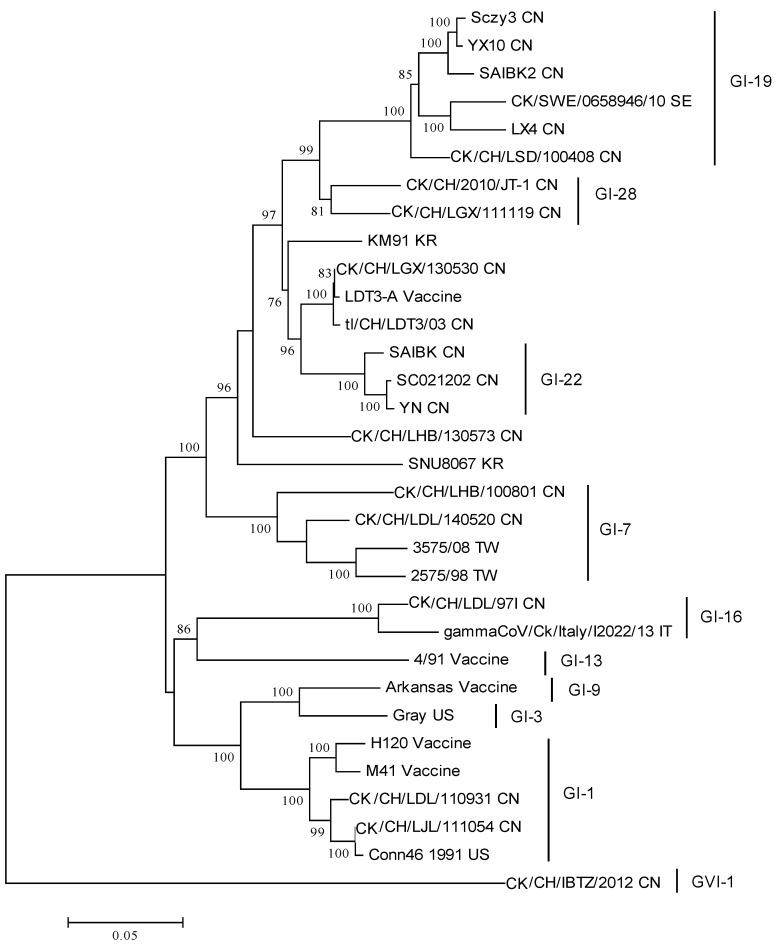
Phylogenetic analysis of the complete *S1* gene of the IBV variants reviewed in this study and reference strains. The phylogenetic tree was constructed using MEGA version 6 by the neighbor-joining method (bootstrapping for 1000 replicates, bootstrap value >70%). CN: China; IT: Italy; KR: Korea; SE: Sweden; TW: Taiwan; US: United States.

**Figure 2:**
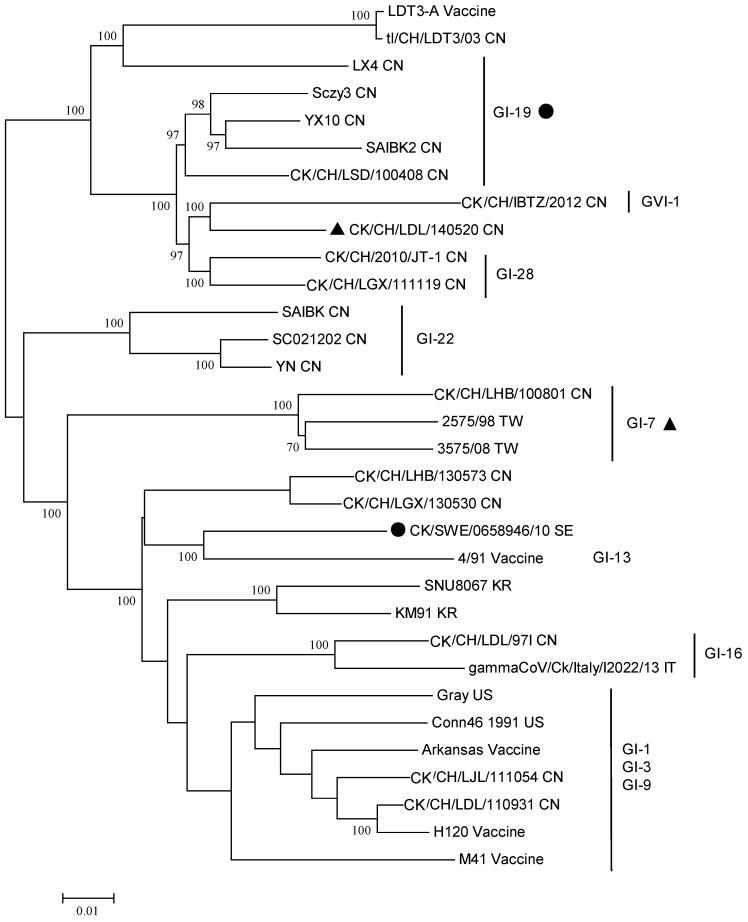
Phylogenetic analysis of the full-length genome of the IBV variants reviewed in this study and reference strains. The strains with a symbol belong to the lineage with the same symbol when the *S1* gene is analyzed. The phylogenetic tree was constructed using MEGA version 6 by the neighbor-joining method (bootstrapping for 1000 replicates, bootstrap value >70%). CN: China; IT: Italy; KR: Korea; SE: Sweden; TW: Taiwan; US: United States.

Phylogeny of the reference strains and the IBV variants reviewed in this article is shown in Figure 1, based on classification scheme described by Valastro et al. [18]. The strain name, GenBank accession number and geographic origin are listed in the Supplementary Table S1. Interestingly, some IBV variants are not consistently classified in the same groups or lineages when analyzed using the full-length genomes (Figure 2). For instance, the *S1* gene of the CK/CH/LGX/111119 belongs to the GI-7 lineage, however, the full genome of this variant appears more related to the GVI-1 or GI-28 lineage, suggesting the genome may comprise segments originated from other lineages, e.g., inter-lineage recombination. Similar findings are also observed in the strain CK/SWE/0658946/10. Thus, examining the full-length genomes of novel IBV strains and understanding their contextual sequences are of equal importance.

In studies correlating genotypes, serotypes and pathogenic types, genotyping and serotyping were consistent in most cases. In addition, according to Jackwood et al. [106] and Lee et al. [107], various serotypes defined by neutralization were more than 10% genetically different, with some exceptions [23,26,27,34,35]. Cavanagh et al. [3] noted that only a few amino acid substitutions, resulting in 2% sequence divergence in the *S1* gene, can alter the serotype, and determining the pathogenic type according to genotype is risky. Evidence indicates that non-structural proteins, accessory proteins, and nucleocapsid proteins can influence viral replication or host-antigen interactions in different phases, thereby influencing virulence. Examples include inhibition of the signal transducer and activator of transcription 1 (STAT 1) signaling pathway, interferon production [108,109], and modulation of viral transcriptional and translational levels [110]. For protectotype identification, determining serotypes by virus neutralization is not sufficient, since systemic antibody, mucosal, and cellular immune responses must be considered together to evaluate immune protection. When investigating the pathogenicity of IBV variants, a tendency for the virus to infect more extensive tissues was found. IBV was first identified as a respiratory system pathogen, and then became pathogenic against the renal and reproductive systems, and the proventriculus. Among these, proventriculus infection greatly decreases the feed conversion rate, and infection of the reproductive system, such as the oviduct, results in the so-called false layer syndrome, resulting in large economic losses in the poultry industry.

Over the past decade, IBV variants have continuously emerged, and most have been isolated from chicken flocks receiving primary or boost immunizations. Different efficacies of the same types of vaccine immunizations created in both the laboratory and in commercial settings serve as a warning [97] that an immune strategy urgently needs to be refined to choose appropriate vaccines and effective immune routes. Many of the IBV variants described in this review have not emerged via accumulated point mutations, but rather by recombination with other existing strains. While the existing strains may have been discovered decades ago and have not circulated in their original regions since that time, they have not vanished and are still evolving, seeking an appropriate environment to cause outbreaks. This worldwide recombination may result from the continuous expansion of global poultry product trade and the migration of people and wild birds, carrying IBV antigens all around the world, and thus provides evidence that environmental selection deeply influences IBV variation. Facing this complex situation, we must admit the importance of developing potent IBV vaccines to ensure biosecurity and global cooperation. This review of IBV variants has practical value for understanding their phylogenetic relationships and epidemiology from both regional and worldwide viewpoints.

## Figures and Tables

**Table 1:** Genotypes, serotypes and pathotypes of the described infectious bronchitis virus (IBV) variants.

IBV Strain	Genotype	Lineage *	Geographic Origin	Immunization History	Arose From	Serotype or Protectotype	Viral Distribution	Mortality	References
CK/CH/LGX/111119	Novel	GI-28	China	H120 and 4/91 vaccinated yellow broiler	LX4, LDL/091022 and an unknown strain	Novel, different from Mass, 4/91, LX4, LDL/97I, and TW-I	Kidney, bronchi, cecal tonsils, oviduct	30% in one-day-old specific-pathogen-free (SPF) white leghorn chickens with 10^6^ 50% embryo infective dose (EID_50_)	[20]
CK/CH/2010/JT-1	Novel	GI-28	China	H120 and 4/91 vaccinated broiler	QX, LSC/ 99I, LDT3/03 and 4/91	Novel, different from Mass and 4/91	Tracheal, lung, kidney	43.75% in three-day-old SPF white leghorn chickens with 10^5^ EID_50_	[21]
SAIBK2	Grouped in the most predominant genotype in China with YN, Sczy3, etc.	GI-19	China	H120 vaccinated layer chickens	YX10, YN, and Mass	N.D.**	Kidney	50% in one-day-old SPF chickens with 10^5^ EID_50_	[22]
CK/CH/LDL/110931 CK/CH/LHB/130573	Mass	GI-1	China	H120 vaccinated	H120-like vaccine strain and other types of viruses	Novel, different from H120, Conn and LDT3	N.D.	N.D.	[23]
YN	Novel	GI-22	China	H120 vaccinated broiler	N.D.	N.D.	Tracheal, lung, kidney, bursa, ovary, oviduct	65% in 30-day-old SPF chickens with 10^5^ EID_50_ 40.5% in 21-day-old commercial laying hens with 10^6.5^ EID_50_	[24,25]
GD	TW-like	GI-7	China	Mass-type vaccinated broiler	QX-like (S2, E, M and N) and TW-like (S1)	QX	Tracheal, lung, kidney, bursa, spleen, proventriculus	40% in 21-day-old SPF chickens with 10^6^ EID_50_	[26,27]
CK/CH/LDL/140520	TW-1	GI-7	China	H120 vaccinated	LX4 and Taiwan group 1 (TW-1)	Different from Mass, LDT3, 4/91, and Conn	Tracheal, kidney, oviduct	30% in one-day-old SPF chickens with 10^5.5^ EID_50_	[28]
CK/CH/LGX/130530	LDT3	Unknown	China	H120 and Ma5 vaccinated broiler	H120 (nsp1-14) and LDT3/03 (nsp14-N)	LDT3-A vaccine provide protection while H120 not	Tracheal, kidney	0% in one-day-old SPF chickens with 10^5^ EID_50_	[29]
CK/CH/LJL/111054	Conn	GI-1	China	H120 vaccinated layer	Mass type and Conn type	N.D.	N.D.	0% in one-day-old SPF chickens with 10^5.5^ EID_50_	[30]
CK/CH/IBTZ/2012	N.D.	GVI-1	China	H120 vaccinated layer	LX4 and an unknown strain in *S* gene	N.D.	N.D.	N.D.	[31]
Sczy3	LX4	GI-19	China	Unknown	LX4 (major) and H120 (minor)	N.D.	N.D.	N.D.	[32]
SAIBK	Novel	GI-22	China	Unknown	SC021202 (major) and H120	4/91-like			[33]
3575/08	TW-I	GI-7	Taiwan	TW-I vaccinated broiler	TW-I strain with point mutations	Novel, distinct with Mass or other local types	Trachea, proventriculus, kidney, bursa, oviduct	83.3% in one-day-old SPF chickens with 10^6^ EID_50_	[34]
JP/Wakayama/2003 JP/Iwate/2005	4/91	GI-13	Japan	Without 4/91 immunization	N.D.	4/91, JP/Iwate/2005 can be protected by 4/91 and JP-II strain.	Respiratory tract	15.4% in one-day-old SPF chickens with 10^4^ EID_50_ of JP/Wakayama/2003	[35]
SNU8067	Korean group I	GI-15	South Korea	Unknown	Korean group II strain KM91 (except S1, 3a,3b) and Korean group I (S1)	KM91 and M41 inactivate vaccine protect 70% from oviduct lesion	Oviduct	N.D.	[36]
IBV/MN IBV/RA IBV/TU	Italy 02	Unknown	Morocco	Unknown	N.D.	N.D.	Respiratory sign without renal lesion	0% in one-day-old SPF chickens with 10^3.5^ EID_50_	[37]
IBV-EG/1212B-2012 IBV-EG/IBV1-2011	Egy/Var II Egy/Var I	Unknown	Egypt	Unknown	Egyptian variant strains or Middle East strains with Israeli strains	N.D.	Tracheal, kidney	50% to 40% in one-day-old SPF chickens with 10^5^ EID_50_	[38,39]
IBV/Cal99 variant/07	Respiratory pathogenic Cal99 genotype	GI-9	US	Game chickens without IBV vaccination	N.D.	N.D.	Trachea, lung, kidney, salivary glands, small intestine	N.D.	[40]
IBV/Brazil/2005/USP-10	Brazilian type	Unknown	Brazil	Mass type vaccinated broiler	N.D.	N.D.	Respiratory tissue, kidney, intestine and testis	None in 26-day-old SPF chickens and male Ross broilers	[41]
γCoV/CK/Italy/I2022/13 IBV/FARM 1 IBV/FARM 5	Q1	GI-16	Italy	H120 vaccinated broiler	Q1 and unknown minor parent strains	Q1 type	Trachea, lungs, kidneys, proventriculus	N.D., Ranging from 4.1% to 9.8% in broiler outbreak	[42,43]
KG3P	QX-like	GI-19	England	793B vaccinated Broiler	N.D.	N.D.	Trachea, lungs, kidneys, proventriculus	16.7% in SPF chickens with 10^4^ TOC_50_	[44]
N1/08	Australian group 2	GIII-1	Australia	Unknown	Australian group 2 and group 3 strains in S	N.D.	Trachea, cecal tonsils	None in 14-day-old SPF chickens with 10^5.5^ EID_50_	[45,46]

* Classification based on the *S1* gene, according to Valastro et al. [18]; ** N.D.: Not done.

## Supplementary Materials

Supplementary materials can be found at www.mdpi.com/1422-0067/18/10/2030/s1.
