# Supplementary material for: Infectious Bronchitis Virus Variants: Molecular Analysis and Pathogenicity Investigation
Source: Int J Mol Sci. 2017 Sep 22;18(10):2030. doi: 10.3390/ijms18102030 (PMC5666712; doi:10.3390/ijms18102030)
Supplement: Supplementary file 1 [file ijms-18-02030-s001.pdf]

Supplementary Table S1. IBV strains used for the phylogenetic analyses. The strain name, GenBank accession number and geographic origin are listed.

Strain	GenBank Accession No.	Geographic Origin	Strain	GenBank Accession No.	Geographic Origin
4/91 Vaccine	KF377577	-	LX4	AY338732	China
Arkansas Vaccine	GQ504721	-	SAIBK	DQ288927	China
H120 Vaccine	FJ888351	-	SAIBK2	KU317090	China
LDT3-A Vaccine	KR608272	-	SC021202	EU714029	China
M41 Vaccine	DQ834384	-	Sczy3	JF732903	China
CK/CH/2010/JT-1	KU361187	China	tl/CH/LDT3/03	KT852992	China
CK/CH/IBTZ/2012	KF663559	China	YN	JF893452	China
CK/CH/LDL/97I	JX195177	China	YX10	JX840411	China
CK/CH/LDL/110931	KJ425485	China	2575/98	DQ646405	Taiwan
CK/CH/LDL/140520	KP790143	China	3575/08	KX266757	Taiwan
CK/CH/LGX/111119	KX640829	China	KM91	JQ977698	South Korea
CK/CH/LGX/130530	KP343691	China	SNU8067	JQ977697	South Korea
CK/CH/LHB/100801	JF330898	China	CK/SWE/0658946/10	JQ088078	Sweden
CK/CH/LHB/130573	KJ425496	China	γCoV/CK/Italy/I2022/13	KP780179	Italy
CK/CH/LJL/111054	KC506155	China	Conn46 1991	FJ904719	United States
CK/CH/LSD/100408	KX236007	China	Gray	GU393334	United States
